# High-yield production in *Escherichia coli* and convenient purification of a candidate vaccine against SARS-CoV-2

**DOI:** 10.1007/s10529-022-03298-z

**Published:** 2022-09-26

**Authors:** Giulia Maltoni, Lorenzo Scutteri, Francesca Mensitieri, Fabrizio Dal Piaz, Alejandro Hochkoeppler

**Affiliations:** 1 Department of Pharmacy and Biotechnology, University of Bologna, Viale Risorgimento 4, 40136 Bologna, Italy; 2 CSGI, University of Firenze, Via della Lastruccia 3, 50019 Sesto Fiorentino, Italy; 3 Department of Medicine, University of Salerno, Via Giovanni Paolo II 132, 84084 Fisciano, Italy

**Keywords:** Candidate vaccine, COVID-19, CRM197, *Escherichia coli*, Inclusion bodies

## Abstract

**Objectives:**

The aim of the present work was to identify a time-saving, effective, and low-cost strategy to produce in *Escherichia coli* a protein chimera representing a fusion anti-SARS-CoV-2 candidate vaccine, consisting of immunogenic and antigenic moieties.

**Results:**

We overexpressed in *E. coli* BL21(DE3) a synthetic gene coding for CRM197-RBD, and the target protein was detected in inclusion bodies. CRM197-RBD was solubilized with 1 % (w/v) of the anionic detergent N-lauroylsarcosine (sarkosyl), the removal of which from the protein solution was conveniently accomplished with Amberlite XAD-4. The detergent-free CRM197-RBD was then separated from contaminating DNA using polyethylenimine (PEI), and finally purified from PEI by salting out with ammonium sulfate. Structural (CD spectrum) and functional (DNase activity) assays revealed that the CRM197-RBD chimera featured a native and active conformation. Remarkably, we determined a yield of purified CRM197-RBD equal to 23 mg per litre of culture.

**Conclusions:**

To produce CRM197-RBD, we devised the use of sarkosyl as an alternative to urea to solubilize the target protein from *E. coli* inclusion bodies, and the easy removal of sarkosyl by means of Amberlite XAD-4.

**Supplementary Information:**

The online version contains supplementary material available at 10.1007/s10529-022-03298-z.

## Introduction

The COVID-19 (COrona VIrus Disease 19) outbreak has been a major health concern in the last two years, globally infecting more than 5.4 × 10^8^ individuals and causing 6.32 × 10^6^ deaths as of June 2022 (World Health Organization, COVID-19 Dashboard). To tackle the pandemic, quite a number of vaccines were developed, with some members of this repertoire receiving the authorization for emergency use. Remarkably, most of these vaccines were raised against the spike protein of SARS-CoV-2 (Severe Acute Respiratory Syndrome CoronaVirus 2, the causative agent of COVID-19). In particular, the spike protein was targeted by means of mRNA or recombinant virus technologies, and the first subunit vaccine was recently approved (Thibault et al. 2022). Irrespective of the strategy used to contain the COVID-19 pandemic, a major concern is the emergence of SARS-CoV-2 mutants (Thibault et al. 2022). Accordingly, the occurrence of present and of potentially future SARS-CoV-2 variants demands for vaccination on a global scale, requiring the production at low cost of an efficient vaccine.

When subunit vaccines are considered, their production by overexpression in *Escherichia coli* represents a convenient and time-saving strategy. Moreover, it is of importance to note that the use of synthetic genes coding for natural or artificial proteins is a well-established technique for *E. coli* expression systems, where codon optimization leads to a high probability of obtaining the desired overexpression (Elena et al. 2014). Furthermore, synthetic genes can be designed to code for protein chimeras, e.g. for a conjugate vaccine consisting of immunogenic and antigenic moieties. Therefore, the production of proteins in *E. coli* to be tested as candidate vaccines against SARS-CoV-2 was attempted. In particular, the Receptor-Binding-Domain (RBD) of spike protein was overexpressed using *E. coli* BL21(DE3) or its derivative Rosetta(DE3). The target protein was then recovered from inclusion bodies and subsequently purified (Fitzgerald et al. 2021; Gao et al. 2021). However, it has been reported that the RBD of spike protein features poor immunogenicity (Tan et al. 2021), most probably because of its low molecular mass. As an alternative to using spike protein RBD as a candidate vaccine, three different protein chimeras consisting of a carbohydrate binding module from *Thermotoga maritima* fused to spike protein fragments were produced at high levels in *E. coli*. Remarkably, a yield of purified protein equal to 122 mg/L was obtained with the best performing fusion (McGuire et al. 2022). We have recently shown that a protein chimera consisting of the amino acids 1–388 of CRM197 (a non-toxic variant of diphtheria toxin, Uchida et al. 1973) fused to the RBD of spike protein (residues 389–611) can be successfully overexpressed in *E. coli* and purified from inclusion bodies by conventional methods (Bellone et al. 2021). A quite similar strategy to produce a candidate vaccine was reported shortly thereafter (Krynina et al. 2021).

Here we report a major improvement in the production process of the CRM197-RBD chimera. In particular, we show here a procedure that provides a high yield of CRM197-RBD in a limited number of steps, along with a functional characterization of the purified product.

## Materials and methods

### Protein overexpression

The synthetic gene previously described was used to overexpress the CRM197-RBD protein chimera in *E. coli* BL21(DE3), transformed with the pET9a-CRM197RBD construct (Bellone et al. 2021). Overexpression of the target protein was performed according to the procedure reported by Park et al. (Park et al. 2018). Cells containing the overexpressed protein were collected by centrifugation, and the cell pellets were stored at −20 °C until used.

### Protein purification

The cell pellets were gently resuspended in Falcon tubes with 40 ml of 20 mM Tris/HCl, pH 7.5, and the cells were then lysed by sonication (four 2 min cycles, with each cycle consisting of pulses of 15 s spaced by pauses of the same time length). Samples were kept on ice during sonication, and the power output was set at 18 W. The extract was centrifuged at 13,000×*g* (20 min, 4 °C), and the pellet containing the inclusion bodies was resuspended in 25 mM Tris/HCl, 3 mM cysteine, 0.3 mM cysteine and 1% (w/v) N-lauroylsarcosine (also denoted as sarkosyl), pH 8.0. The suspension was incubated for 15 h under mild shaking, and then centrifuged at 13,000×*g* (20 min, 4 °C). The supernatant, usually 40 ml in volume, was supplemented with 5 mM EDTA and 1.875 g of Amberlite XAD-4, and incubated under mild shaking at room temperature for 3.5 h. The resin was then decanted and discarded, and 1.875 × *g* of Amberlite XAD-4 was added to the supernatant, which was incubated for 1 h under mild shaking at room temperature. After decanting and discarding the resin, the resulting solution was supplemented with 1 M NaCl, and incubated for 15 min. Then, 0.35% (w/v) polyethylenimine (PEI) was slowly added to the sample, which was incubated for 15 min at room temperature. The solution was then centrifuged (13,000×*g*, 10 min, 4 °C), and 2.9 M ammonium sulfate was gradually added to the supernatant. Finally, the turbid protein solution was centrifuged (10,000×*g*, 20 min, 4 °C), the pellet was resuspended in PBS and given three dialysis cycles with the same buffer. Aliquots of the dialyzed protein were stored at −20 °C until used. Protein concentration was determined by the Bradford assay (Bradford 1976).

### Activity assays

The DNase activity of CRM197-RBD was assayed spectrophotometrically (Kunitz 1950), using calf thymus DNA as substrate in the presence of 50 mM Tris/HCl (pH 7.6), 2.5 mM MgCl_2_, 2.5 mM CaCl_2_, 40 µg DNA ml^−1^, and enzyme. One Kunitz Unit was defined as the amount of enzyme producing an increase in Absorbance of 0.001 (at 260 nm) per minute per ml at 25 °C and pH 7.6.

### Mass spectrometry

To verify the identity of the proteins, spots were excised from gels and underwent trypsin in-gel digestion as previously reported (Shevchenko et al. 2007). The resulting peptides were analysed by LC–MS/MS using a Q-Exactive instrument (Thermo-Fisher Scientific, Waltham, MA, USA) equipped with a nano-ESI source coupled with a nano-Ultimate capillary UHPLC (Thermo-Fisher Scientific) as reported elsewhere (Conte et al. 2012).

To define the disulphide-bond pattern of the CRM197-RBD chimera, trypsin digestion was performed in solution incubating 30 µg unreduced protein with 50 ng of trypsin at 37 °C overnight. The peptide mixture accordingly obtained was split in two and one aliquot was incubated with 25 mM DTT at 40 °C, overnight. Both samples were acidified to a final concentration of 0.1% formic acid and analysed by LC/MS/MS.

### Dynamic light scattering

Dynamic light scattering experiments were performed with a Malvern Panalytical (Malvern, UK) Zetasizer Nano ZS system. All measurements were recorded at 25 °C. Scattering was evaluated at an angle of 173 degrees.

### Circular dichroism

CD spectra were recorded over the 200–250 nm wavelength interval at a scan rate of 50 nm/min, using a Jasco J-810 spectropolarimeter. Protein samples were in PBS buffer, and the band width was set at 1 nm. Twenty-four scans per sample were acquired and averaged.

### Surface plasmon resonance

The binding of CRM197-RBD to angiotensin-converting enzyme 2 (ACE2) was analysed using a Biacore 3000 instrument (Cytiva) as previously described (Bellone et al. 2021). ACE2 surfaces were prepared on research grade CM5 sensor chips (Cytiva) by immobilizing the protein (50 μg ACE2 ml^−1^ in 40 mM CH_3_COONa, pH 5) using a standard amine—coupling protocol; this procedure led to an observed density of 30 kRU. CRM197-RBD was equilibrated and diluted in 0.1 M HEPES, 1.5 M NaCl, 0.03 M EDTA, 0.5% v/v surfactant P20 (pH 7.4), to obtain sample at four different concentrations (2, 4, 8, and 32 nM, respectively).

Binding experiments were performed at 25 °C with 120 s of association time and 300 s of dissociation time (flow rate 30 µL/min). The observed curves were fitted to a single-site interaction model, yielding a single K_D_. Sensorgrams elaboration was performed using the BIAevaluation software, provided by Cytiva.

### ELISA assays

CRM197-RBD (5 µg protein ml^−1^, in PBS) was adsorbed on 96-microwell plates (BRANDplates® cellGrade™, Lot 489,851) overnight at 4 °C. The wells were then blocked with PBS containing 0.1% Tween 20 (v/v) and 2% (w/v) BSA for 90 min, at room temperature (RT). The blocked wells were subsequently subjected to 4 washes with PBS containing 0.1% Tween 20 (PBS/T). Appropriate dilutions in PBS of the primary antibody (rabbit polyclonal anti-SARS-CoV-2 spike protein RBD domain, Merck Millipore, Lot Q3746488) were then dispensed to the wells containing immobilized CRM197-RBD and incubated for 120 min at RT. Finally, after 4 consecutive washes with PBS/T the secondary antibody (anti-rabbit IgG-peroxidase, Merck Millipore) was added to the wells, and incubated for 120 min at RT. After 4 washes with PBS/T, the reaction catalyzed by peroxidase was determined in the presence of 2,2’-Azino-bis(3-ethylbenzothiazoline-6-sulfonic acid) and H_2_O_2_ (at 9.7 mM and 0.01%, respectively), and determining the Absorbance changes at 600 nm using a Bio-Rad 550 Microplate Reader. To perform avidity tests, the wells containing immobilized CRM197-RBD associated to the primary antibody were washed with PBS or with PBS supplemented with 8 M urea. Accordingly, the avidity index was expressed as the ratio of the ELISA response obtained with the untreated samples over the output observed with samples exposed to 8 M urea.

## Results and discussion

**Fig. 1:**
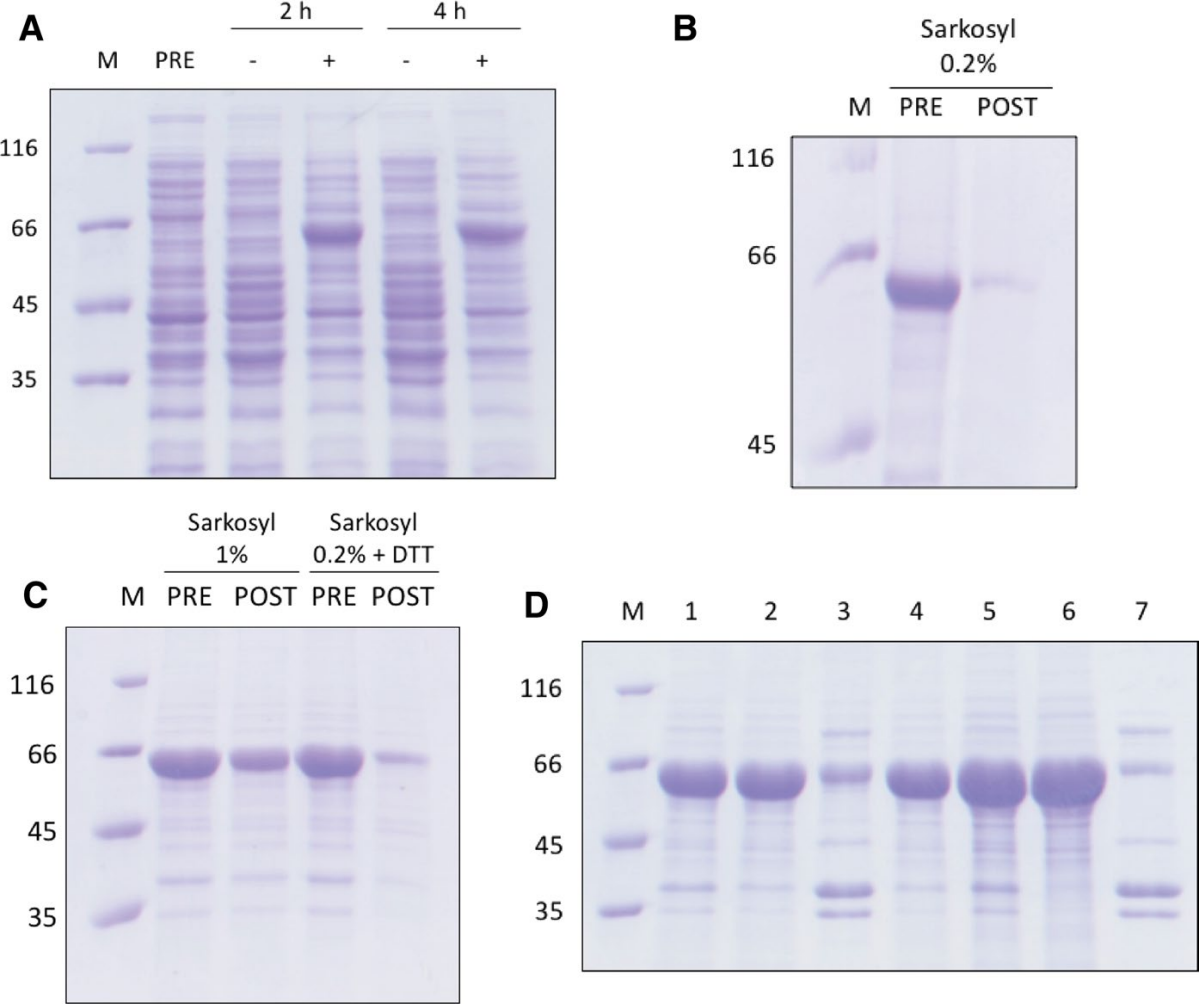
Overexpression of CRM197-RBD in *Escherichia coli*, solubilization from inclusion bodies and refolding. **A** SDS-PAGE of total protein extracts (obtained by boiling cells resuspended with sample buffer) isolated from the pre-culture (PRE) at 37 °C whose Absorbance at 600 nm was equal to 0.6, and from cells collected after 2 or 4 h (h) of further incubation in the absence (-) or in the presence ( +) of 0.5 mM IPTG. **B** Assay of CRM197-RBD solubilization by 0.2% (w/v) sarkosyl. SDS-PAGE was performed with aliquots of a CRM197-RBD suspension incubated with the detergent and subjected (POST) or not (PRE) to centrifugation at 13,000 × *g*. **C** Assay of CRM197-RBD solubilization by 0.2% sarkosyl supplemented with 10 mM DTT, or by 1% of sarkosyl. Other conditions as in Fig. 1B. **D** SDS-PAGE analysis of solubilization and refolding of CRM197-RBD. Partition of the protein chimera incubated with 1% sarkosyl for 15 h (lane 1) into supernatant (lane 2) and pellet (lane 3) after centrifugation at 13,000 × *g*. The supernatant incubated with Amberlite (see Methods) was analysed before (lane 4) and after (lane 5) concentration to 5 mg protein ml^−1^. Partition of the concentrated sample exposed to Amberlite into supernatant (lane 6) and pellet (lane 7) on centrifugation at 13,000 × g is also shown

CRM197 is a variant of diphtheria toxin, bearing the site-specific substitution G52E, which suppresses the ADP-ribosylating activity (Uchida et al. 1973) but does not alter the DNase catalytic action of the toxin (Bruce et al. 1990). Remarkably, CRM197 exposed to formaldehyde features immunogenic properties similar to those of diphtheria toxoid (Porro et al. 1980). Therefore, CRM197 represents a suitable carrier to confer immunogenicity to low molecular mass antigens, and is currently used to produce conjugate vaccines (Rappuoli et al. 2019). We recently reported on the production in *E. coli* of a protein chimera containing a large fragment of CRM197 fused to the RBD of SARS-CoV-2 spike protein (Bellone et al. 2021). However, recovery of this chimera from inclusion bodies and the subsequent purification of this chimera proved quite laborious, consisting of: (i) the solubilization of inclusion bodies with 6 M urea; (ii) an anion-exchange chromatography performed to purify the target protein from contaminating DNA; (iii) a dialysis step to achieve protein refolding; (iv) a final purification step by affinity chromatography carried out with a HiTrap Heparin column. We therefore set out to develop a more convenient procedure to obtain homogeneous CRM197-RBD. In line with a previous report on CRM197 (Park et al. 2018), we first tested the overexpression levels of CRM197-RBD when host cells were subjected to induction for 2 or 4 h with 0.5 mM isopropyl-thio-β-D-galactopyranoside (IPTG), at 37 °C. Interestingly, under both conditions we obtained a high concentration of the target protein when total protein extracts were analysed (Fig. 1A). Further, this high level of CRM197-RBD was detected in inclusion bodies, which we solubilized with a buffer containing 0.2% (w/v) of the anionic surfactant sarkosyl. However, with this detergent concentration, only a minimal amount of the target protein was solubilized (Fig. 1B).

**Fig. 2:**
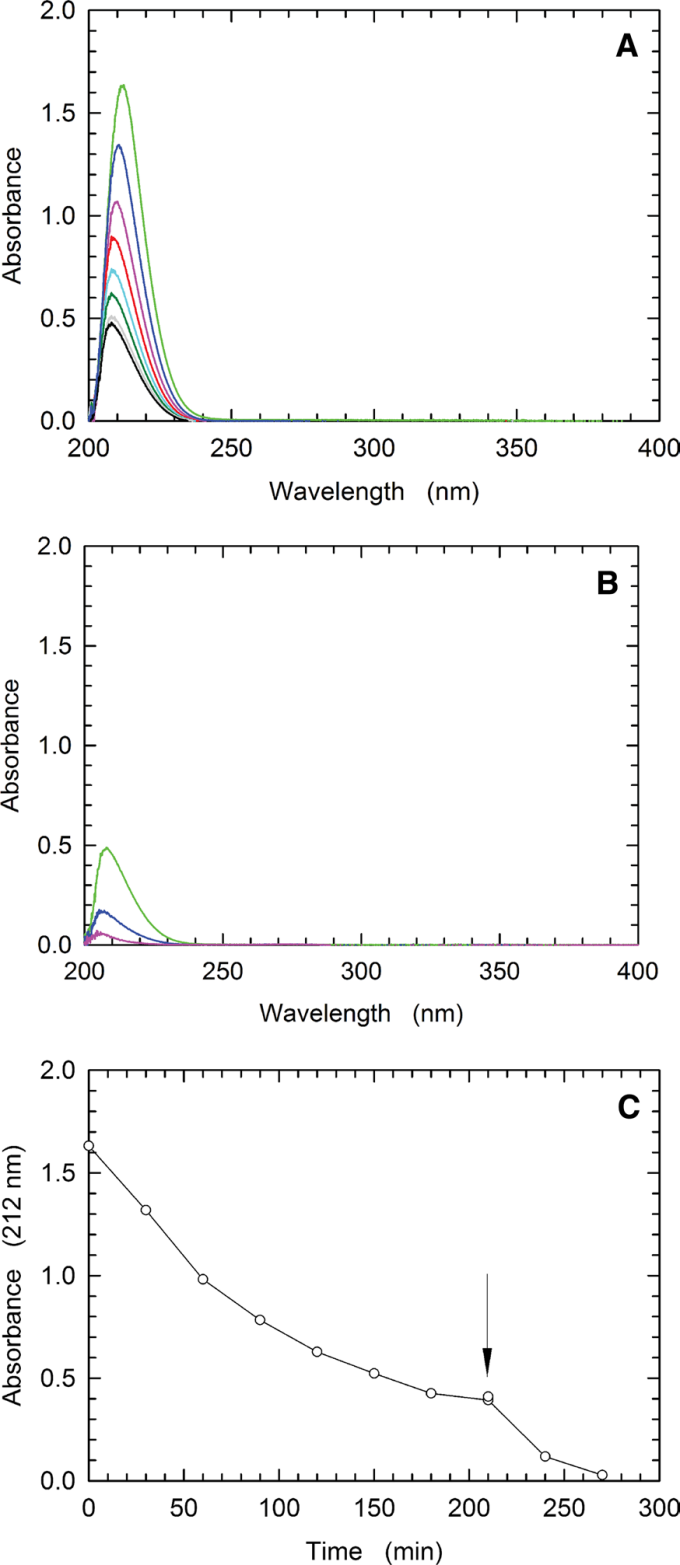
Adsorption of sarkosyl by Amberlite XAD-4. **A** Absorption spectra of a solution containing 50 mM Tris/HCl (pH 8) and 1% (w/v) sarkosyl exposed to 1.875 g of Amberlite XAD-4 at room temperature, under mild shaking conditions. The spectra were recorded before (green line) or 30, 60, 90, 120, 150, 180, and 210 min (blue, magenta, red, cyan, dark green, grey, and black lines, respectively) after the addition of Amberlite to the sample. **B** Absorption spectrum of the sample obtained at the end of the experiment shown in **A** after decanting and discarding Amberlite XAD-4 (green line). The absorption spectra recorded 30 and 60 min (blue and magenta lines, respectively) after addition of 1.875 g of Amberlite to the sample are also shown. **C** Kinetics of sarkosyl adsorption by Amberlite XAD-4 as determined by the Absorbance decrease at 212 nm detected in the spectra reported in **A** and **B**. The arrow indicates when the Amberlite XAD-4 initially added to the sample was replaced with 1.875 g of new resin

We therefore assayed the solubilization of CRM197-RBD supplementing with 10 mM DTT the solubilization buffer, or increasing sarkosyl concentration to 1 %. A satisfactory degree of solubilization was observed with the buffer containing 1 % (w/v) sarkosyl, whereas addition of DTT proved ineffective (Fig. 1C). Accordingly, we solubilized CRM197-RBD from inclusion bodies with a buffer composed of 25 mM Tris/HCl, 5 mM EDTA, 1 % sarkosyl, 3 mM cysteine and 0.3 mM cystine, pH 8.0, with the cysteine/cystine redox couple added specifically to favour formation of the five native disulphide bonds occurring in CRM197-RBD (Bellone et al. 2021). The solubilized CRM197-RBD was then refolded removing sarkosyl by adsorption on Amberlite XAD-4, a crosslinked aromatic polymer. In particular, two cycles of incubation with Amberlite were performed (see Methods). A preliminary test showed that by this means sarkosyl can be completely removed in 4.5 hours from an aqueous solution containing 1 % of the detergent (Fig. 2). Interestingly, the target protein was not adsorbed by Amberlite XAD-4 (Fig. 1D).

**Fig. 3:**
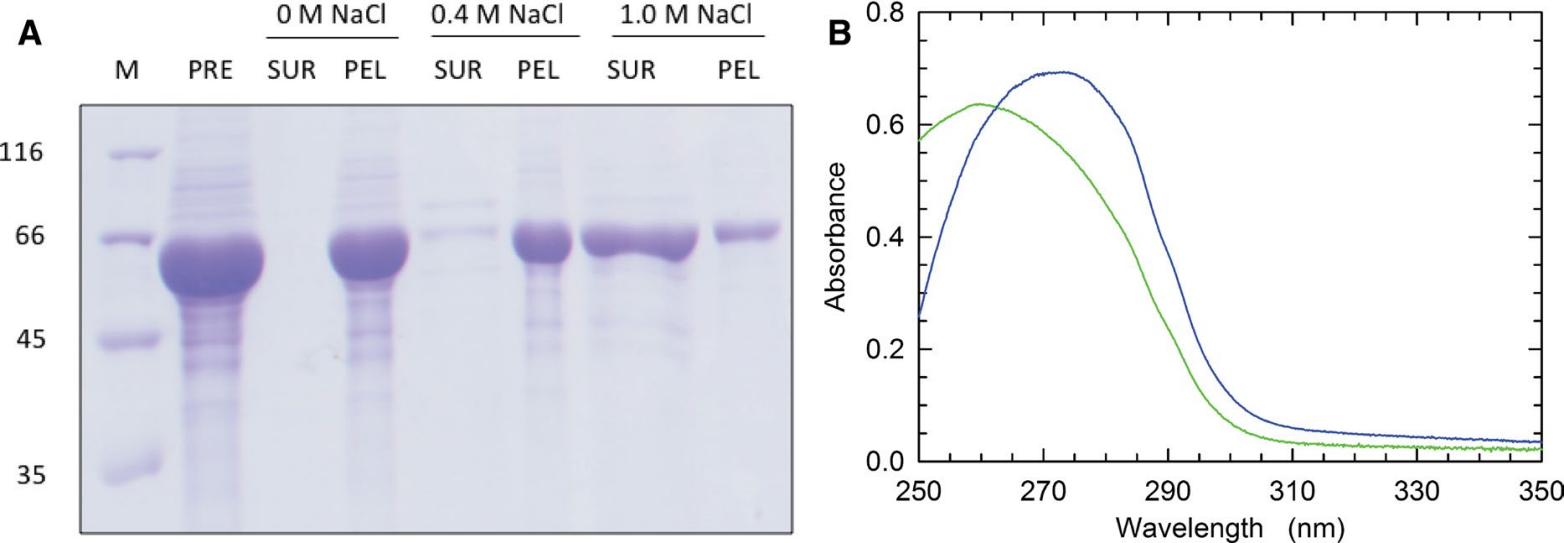
Purification of CRM197-RBD from contaminating DNA. **A** SDS-PAGE of a CRM197-RBD sample contaminated with DNA (PRE) and of aliquots supplemented with 0.35% (w/v) PEI in the absence or in the presence of 0.4 or 1.0 M NaCl. SUR and PEL indicate the supernatant and the pellet obtained after centrifugation of the aliquots at 13,000 × *g*. **B** Absorption spectrum of the CRM197-RBD untreated sample (green line) and of the supernatant obtained by centrifugation after addition of 1 M NaCl and PEI (blue line)

Further, the detergent-free CRM197-RBD was soluble, withstanding concentration to 5 mg of protein ml^-1^ and a subsequent centrifugation at 13,000 x g for 20 minutes, at the end of which only some protein contaminants were sedimented (Fig. 1D). We suggest that this substantial solubility is due to a proper refolding of CRM197-RBD induced by the adsorption of sarkosyl to Amberlite XAD-4. In line with our previous observations (Bellone et al. 2021), we found that CRM197-RBD was tightly bound to DNA, the separation of which from the target protein was accomplished by treatment with polyethylenimine (PEI). After centrifuging the sample containing PEI and 1 M NaCl, most of the target protein was recovered in the supernatant (Fig. 3A), and a spectroscopic analysis showed that the treatment with PEI had been effective in purifying the protein of DNA (Fig. 3B). Remarkably, the final yield of CRM197-RBD estimated by Bradford assay was equal to 23 mg of purified protein per litre of culture, namely 35 % of the protein solubilized from inclusion bodies with 1 % sarkosyl (67 mg).

**Fig. 4:**
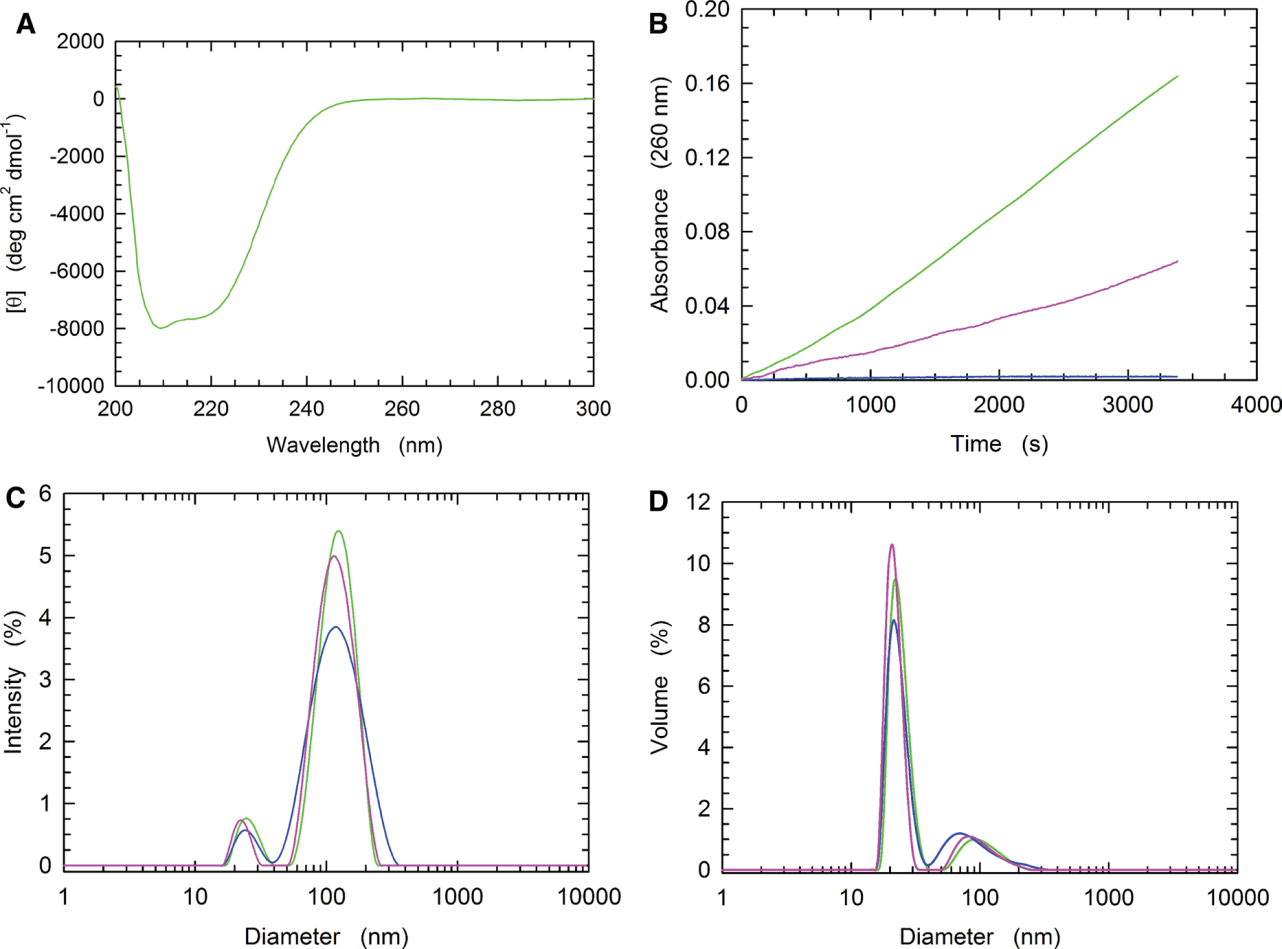
Structural and functional properties of purified CRM197-RBD. **A** Far-UV CD spectrum of CRM197-RBD. **B** DNase activity at the expense of calf thymus DNA exerted by CRM197-RBD (90 µg protein ml^−1^, green line), and by 90 µg of CRM197-RBD (magenta line) overexpressed and purified as previously described (Bellone et al. 2021). The kinetics observed with a control assay performed in the absence of enzyme is also shown (blue line). **C**–**D** Dynamic light scattering experiments carried out with CRM197-RBD. The output of three independent measurements is shown (green, blue, and magenta lines), and is expressed as % of scattering intensity or % of the volume associated with the detected particles

Homogeneous CRM197-RBD was then characterized structurally and functionally. First, we inspected protein folding by circular dichroism (CD), observing a spectrum diagnostic of a native state (Fig. 4A). Moreover, this CD spectrum was quantitatively in agreement with our previous observation obtained with CRM197-RBD solubilized from inclusion bodies with 6 M urea and refolded by dialysis (Bellone et al. 2021), and is quite similar to the CD spectrum of CRM197 produced in *Pseudomonas fluorescens* (Bellone et al. 2021). Further, the correct coupling of cysteines to form the expected disulphide bridges was confirmed by mass spectrometry (Table 1).

**Table 1:** Identification by mass spectrometry of peptides obtained from digestion of CRM197-RBD chimera under not reducing or reducing conditions

Observed Molecular Mass (Da)		Peptide	Theoretical Molecular Mass (Da)
Not reduced	Reduced		
2620.224	2620.225	11–33	2620.227
1384.551	1384.551	40–51	1384.553
1002.427	1002.426	52–59	1002.429
1798.802	1798.803	60–76	1798.801
2362.195	2362.195	105–125	2362.198
2666.323	2666.320	134–157	2666.323
1519.789	1519.785	158–170	1519.788
	1877.748	174–190	1877.749
	1862.920	194–210	1862.920
**3768.651**		**(174–190)-(194–210)**	**3768.653**
2440.194	2440.193	245–264	2440.191
3635.777	3635.775	265–299	3635.774
985.520	985.519	390–398	985.519
	2038.991	399–416	2038.993
1112.540	1112.538	417–425	1112.540
	2315.077	428–456	2315.078
**4352.065**		**(399–416)-(428–448)**	**4352.055**
	853.401	449–456	853.400
	1988.969	457–473	1988.967
898.486	898.487	479–487	898.487
	2208.015	495–514	2208.016
**3059.394**		**(449–456)-(495–514)**	**3059.401**
1217.584	1217.585	515–524	1217.583
	4767.131	537–579	4767.132
**4765.118**		**537–579**	**4765.116**
	1979.090	580–598	1979.091
**3963.998**		**(457–473)-(580–598)**	**3964.015**

The peptide indicated in bold contains a couple of cysteines engaged in a disulfide bridge

To test the folding of CRM197-RBD we also assayed the DNase activity of the protein chimera at the expense of calf thymus DNA. When 90 µg of CRM197-RBD was used in this assay (the final volume of the reaction mixture was 1 ml), we detected 3.2 Kunitz Units (Fig. 4B). In addition, when the activity assay was performed with 90 µg of CRM197-RBD overexpressed and purified according to our previously reported procedure (Bellone et al. 2021) we detected 1.6 Kunitz Units (Fig. 4B).

Therefore, the strategy reported here for the solubilization (from inclusion bodies) and refolding of CRM197-RBD outperforms the corresponding conventional approach that relies on the use of urea and dialysis (Bellone et al. 2021). To test the aggregation state of our preparation of CRM197-RBD, we used dynamic light scattering (DLS). By this means, we observed the occurrence of two peaks, both diagnostic of protein aggregates and featuring diameters of 24.5±1.6 and 125.2±4.1 nm, respectively, with the higher aggregation state dominating the scattering intensity (Fig. 4C). However, when the volume of the particles identified by DLS was considered, those 25 nm in diameter were found to largely prevail (Fig. 4D). It should also be noted that this aggregation form matched that previously identified with CRM197-RBD (solubilized with 6 M urea and subsequently refolded), which featured a diameter equal to 21-27 nm (Bellone et al. 2021).

The RBD of SARS-CoV-2 spike protein is known to associate to the human angiotensin-converting enzyme 2 (ACE2) receptor, with K_D_ values ranging from 1.2 to 44.2 nM (Shang et al. 2020; Walls et al. 2020). To test the binding of CRM197-RBD to ACE2, we performed a Surface Plasmon Resonance (SPR) experiment, the outcome of which translates into a K_D_ equal to 7.5 ± 1.1 nM (Supplementary Information, Fig. S1). This value is comparable to those reported by others (Shang et al. 2020; Walls et al. 2020) and is significantly lower than the value we formerly determined for the complex between ACE2 and CRM197-RBD (Bellone et al. 2021), suggesting that the procedure reported here for the isolation of CRM197-RBD outperforms the method we previously devised (Bellone et al. 2021). Further, to determine the avidity of a rabbit anti-RBD polyclonal antibody exposed to the antigen CRM197-RBD we carried out ELISA assays immobilizing on microwell plates the CRM197-RBD protein chimera. First, we identified 150 µg of primary antibody mL^−1^ (Supplementary Information, Fig. S2A) as a suitable concentration to confer appropriate sensitivity to the avidity test (Dimitrov et al. 2011). Second, we checked the susceptibility of the antigen–antibody complex to the treatment with urea at concentrations from 0 to 8 M (Supplementary Information, Fig. S2B). Finally, by performing 3 independent avidity tests using 8 M urea as the dissociating agent of the antigen–antibody complex, we estimated an avidity index (see Methods) equal to 0.35 ± 0.02. This value is in good agreement with the avidity indexes (ranging from 0.1 to 0.4) evaluated for anti-RBD antibodies evolved after SARS-CoV-2 infection (Tauzin et al. 2022). Overall, the observations we obtained with the SPR experiment and with the ELISA assays indicate that the CRM197-RBD chimera contains a Receptor Binding Domain the conformation of which is comparable to that present in native SARS-CoV-2 spike protein.

## Concluding remarks

Here we report on the production in *Escherichia coli* of a protein chimera, denoted as CRM197-RBD, which is proposed as a candidate vaccine against SARS-CoV-2. In particular, to obtain CRM197-RBD we developed an efficient, time-saving procedure consisting of: (i) a fermentation the length of which is approximately equal to 7 h, including the culture of *E. coli* cells and their induction to overexpress the target protein; (ii) the isolation of non-classical inclusion bodies obtained by cells lysis and a single centrifugation step; (iii) the solubilization of the target protein with sarkosyl, the removal of which from the protein solution is accomplished in 4.5 h by adsorption on Amberlite XAD-4; (iv) the isolation of DNA-free CRM197-RBD by polyethylenimine (PEI) precipitation of the nucleic acid; (v) the separation of PEI from the target protein by salting out with ammonium sulfate. At the end of this procedure, which takes 25–30 h, CRM197-RBD can be dialyzed to obtain the protein in the buffer of choice, e.g. PBS, and free of ammonium sulfate. By this means, we obtained a yield equal to 23 mg of purified protein per litre of culture. It should also be remarked that this yield can be largely increased by production of biomass with appropriate fermenters.

As previously mentioned, the rapid emergence of SARS-CoV-2 variants, such as the present Omicron, is a major concern. The Spike protein of Omicron contains 30 site-specific substitutions, 15 of which reside in the RBD (Cui et al. 2022). Interestingly, none of the eight RBD cysteines are affected by these mutations (Cui et al. 2022), therefore leaving the four disulfide bridges of RBD unaltered. Moreover, the overall tertiary structure of Omicron RBD does not substantially differ from that of its progenitor, except for a significant shift in a α-helix located near the mutated amino acids S371, S373, and S375 (Zhang et al. 2022). Accordingly, and considering the convenience of gene synthesis for rapidly obtaining variants of a coding sequence, the strategy we reported here appears suitable for redirecting the production of vaccines in a timely manner.

Importantly, we also demonstrated that CRM197-RBD is stable at room temperature for 24 h (Bellone et al. 2021). We therefore suggest that the procedure described here will be useful for providing inexpensive vaccines that do not require a cold chain.

## Supplementary Information

Below is the link to the electronic supplementary material.

Supplementary file1 (PDF 645 KB)

## Data Availability

The datasets generated during the current study are available from the corresponding author on reasonable request.
