# Supplementary material for: High-yield production in Escherichia coli and convenient purification of a candidate vaccine against SARS-CoV-2
Source: Biotechnol Lett. 2022 Sep 26;44(11):1313–22. doi: 10.1007/s10529-022-03298-z (PMC9512991; doi:10.1007/s10529-022-03298-z)
Supplement: Supplementary file 1 — Supplementary file1 (PDF 645 KB) [file 10529_2022_3298_MOESM1_ESM.pdf]

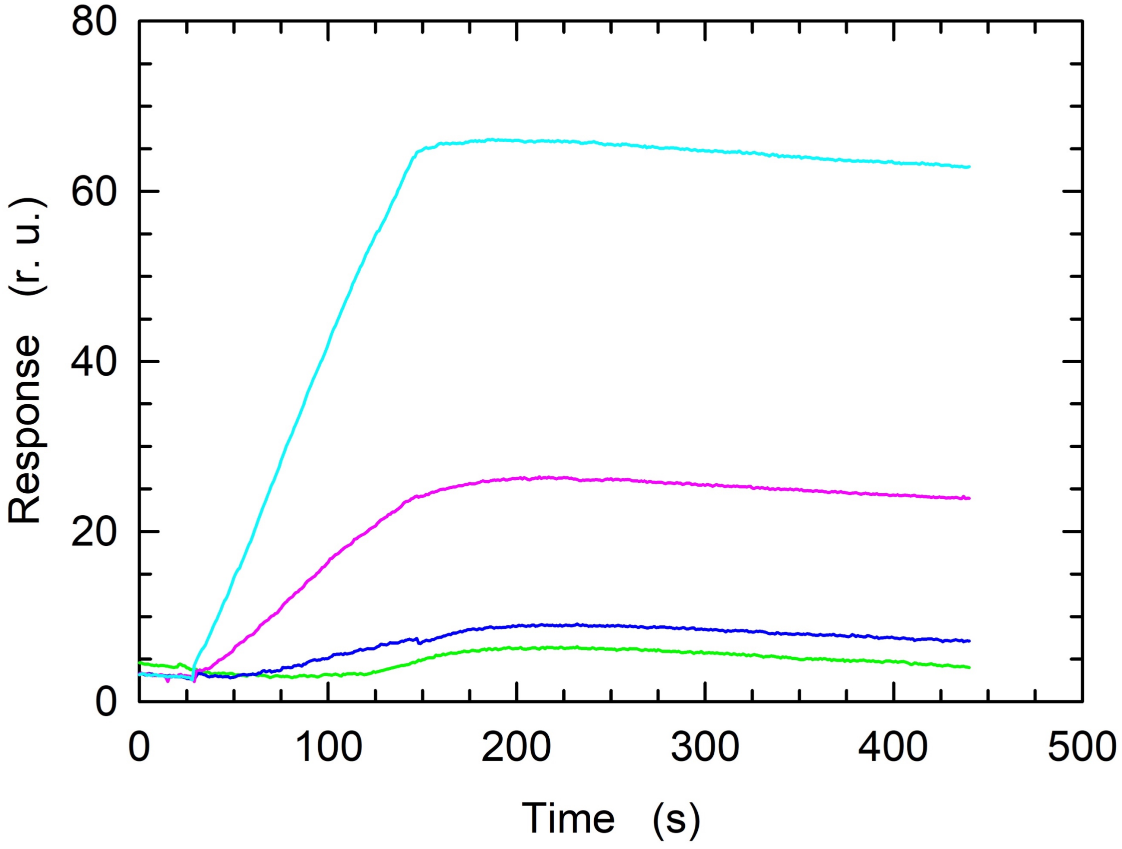

Supplementary Figure S1. Interaction of CRM197-RBD with human ACE2.
Surface plasmon resonance analysis of the interaction of CRM197-RBD with the ACE2 receptor. Sensorgrams observed loading increasing concentration of purified CRM197-RBD (2, 4, 8, and 32 nM, green, blue, magenta, and cyan line, respectively) on a sensor chip modified with immobilized ACE2.

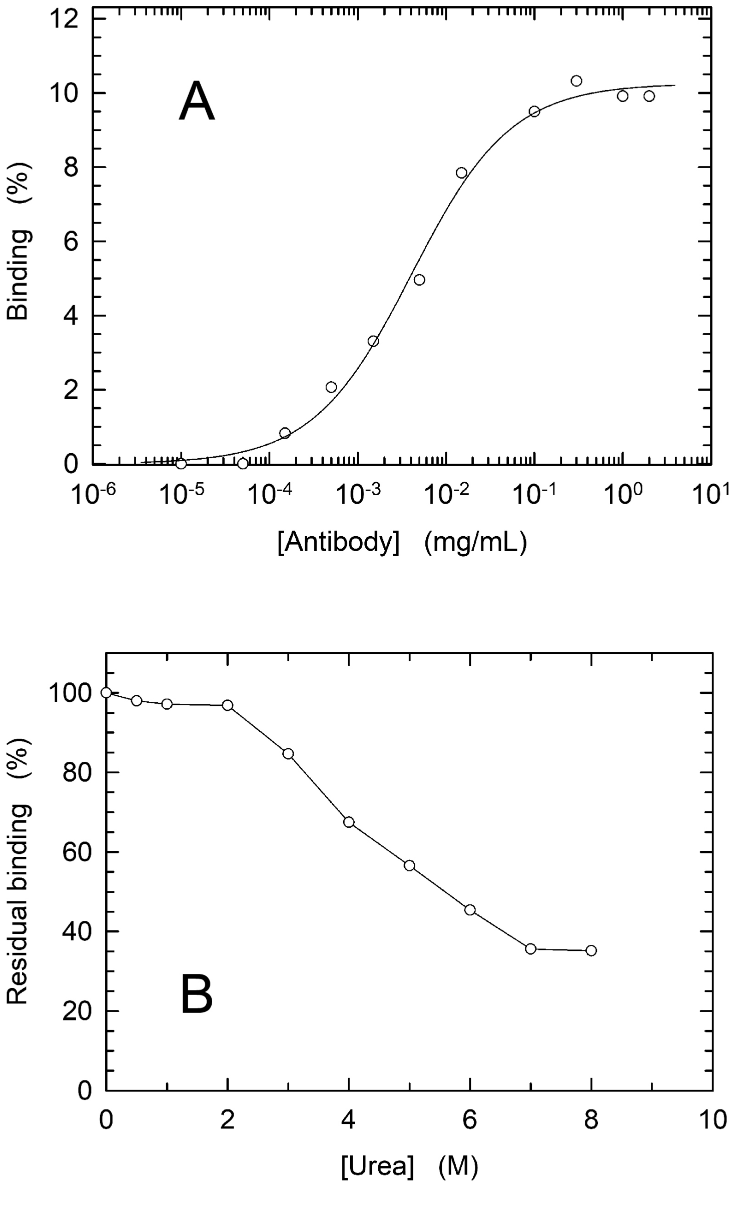

Supplementary Figure S2. Avidity of the complex between CRM197-RBD and a rabbit polyclonal anti-RBD antibody.

(A) Binding of a goat anti-rabbit peroxidase-conjugate secondary antibody as a function of the primary rabbit anti-RBD antibody concentration used to bind the immobilized CRM197-RBD antigen. The extent of binding was calculated by comparing the peroxidase activity detected in the samples (obtained with different concentrations of the primary antibody, and using a constant concentration of the secondary antibody) to the enzyme activity observed in the presence of free secondary antibody at the same concentration used for the samples. (B) Dissociation of the complex between CRM197-RBD and a rabbit polyclonal anti-RBD antibody as a function of urea concentration. Samples were prepared adding the primary rabbit antibody (at 150 µg protein ml^{-1}) to the wells containing immobilized CRM197-RBD. The wells were then subjected with 4 consecutive washings with PBS containing urea at concentrations ranging from 0 to 8 M. Afterwards, all the samples were subjected to 4 further washings with PBS before the addition of the secondary goat anti-rabbit peroxidase-conjugate antibody.
